# Universal mapping of humoral immune response using a versatile high-content and high-density peptide microarray

**DOI:** 10.1186/1742-4690-9-S1-P17

**Published:** 2012-05-25

**Authors:** Ulf Reimer, Nikolaus Pawlowski, Janina Seznec, Tobias Knaute, Paul von Hoegen, Holger Wenschuh, Dan H Barouch

**Affiliations:** 1 Jpt Peptide Technologies, Berlin, Germany; 2 Division of Vaccine Research, Beth Israel Deaconess Medical Center, Harvard Medical School, Boston, MA 02115, USA

## Background

Humoral immune responses are often the hallmark of efficient vaccines. The recent RV144 vaccine trial has turned attention to the stimulation of humoral immune response as a potential mode of action for HIV vaccines. Therefore, detailed monitoring of antibody reactivities in patient specimens before and after vaccination is crucial. The determination of these reactivities on a sub-protein level provides information on the site of antigen/antibody interaction. In contrast to assays relying on whole antigens such as ELISA, peptide microarrays are efficient tools to deliver such information. Besides, complex peptide libraries can cover HIV sequence diversity, a special challenge provided by this virus.

## Materials and methods

Based on the sequence database of LANL a complex peptide library of more than 6500 peptides was generated. The peptides were synthesized and printed onto glass slides.

Initial incubations with serum samples of non-human primates from vaccination studies were performed and evaluated.

## Results

The peptides span the immunogenic regions of the HIV proteome including full-length ENV, NEF and fractions of GAG, POL, TAT, REV and VIF and allow an overall coverage above 50 % of all HIV sequences. Each clade (A, B, C, D, G, CRF1 and CRF2) is represented by at least one sequence. Additional sequences were added to improve coverage.

Experimental data for serum samples from vaccination trials allow the identification of antibody reactivities following vaccination. The representation of different clades allows a detailed evaluation of specificity for the raised antibodies.

## Conclusion

High-density high-content peptide microarrays can tackle the tremendous sequence diversity of HIV and deliver information on clade-specific antibody response. This enables monitoring of humoral immune response in HIV patients independent of geographical origin and to study a broad range of different vaccines. The results can shed light on the underlying protective mechanisms of vaccinations.

